# Associations Between Exercise Experiences in the Home Environment During Early Childhood and Motor Skill Development in Preschool Children: A Longitudinal Follow-Up Study

**DOI:** 10.3390/children13070947

**Published:** 2026-07-19

**Authors:** Shiho Kobayashi, Keiko Abe, Kozo Tomiyama

**Affiliations:** 1 Faculty of Education, Osaka Seikei University, Osaka 533-0007, Japan; abe-ke@osaka-seikei.ac.jp; 2 School of Sport Sciences, Osaka University of Health and Sport Sciences, Osaka 590-0459, Japan; tomiyama@ouhs.ac.jp

**Keywords:** early childhood, motor skills, exercise experiences, physical activity, home environment, longitudinal follow-up study

## Abstract

**Highlights:**

**What are the main findings?**

- Initial motor skill gaps in boys with limited early exercise experience appeared to diminish over time in specific skills (e.g., double-leg continuous jumps); however, these catch-up trends should be interpreted cautiously as exploratory observations.
- The association between early exercise experiences and motor development exhibits distinct gender differences during the preschool years.

**What are the implications of the main findings?**

- Preschool environments may serve a vital complementary function, potentially helping to narrow developmental gaps associated with limited physical activity at home.
- Fostering pediatric motor development requires a collaborative approach that integrates both home and early educational settings to address gender-specific needs.

**Abstract:**

**Background/Objectives:** Early childhood is a critical period for physical development, yet the long-term impact of initial exercise experiences on specific motor skills remains underexplored. This study adopted a longitudinal follow-up design to examine the development of specific motor skills, while early exercise experiences were assessed retrospectively. We examined differences by age, sex, and skill type. **Methods:** Participants were drawn from a single private preschool in Japan. Early exercise experiences defined as the frequency of physical play and active movement opportunities in the home environment were retrospectively categorized as abundant or limited based on parental reports. Five motor skills (25 m dash, standing long jump, tennis ball throw, double-leg continuous jump, and plank duration) were measured longitudinally in children at ages 3, 4, and 5. **Results:** Among boys, the abundant group demonstrated significantly superior performance in four of the five motor skills, excepting the tennis ball throw. Notably, a significant interaction between age and early exercise experiences was observed in boys’ double-leg continuous jump (*p* = 0.041, $\eta_{p}^{2}$ = 0.044); the initial performance gap at age 3 substantially diminished by age 5, illustrating a catch-up effect. Conversely, no consistent differences based on early exercise experiences were observed among girls across any motor skills. **Conclusions:** Motor skill development during early childhood is highly skill-specific and sex-dependent. The observed catch-up effect in double-leg continuous jumps, although requiring cautious interpretation due to the observational nature of this study, suggests that preschool environments may play a vital role in bridging early developmental gaps. Ultimately, this study highlights the crucial, complementary roles of both home and early childhood education settings in maximizing motor development.

## 1. Introduction

In recent years, concerns have emerged regarding the physical fitness and motor skills of Japanese children and students from a medium- to long-term perspective. The 2025 Survey on Physical Fitness and Motor Skills indicated that, although total physical fitness scores for both boys and girls in elementary and junior high schools improved compared with the previous year, they have not yet fully recovered [1]. These trends are often perceived as issues arising during the school-age years; however, it is also possible that the quality of exercise experiences in earlier developmental stages is associated with these outcomes.

Early childhood is widely recognized as a critical period of rapid physical growth during which basic motor skills develop quickly. The theoretical rationale for focusing on this period is supported by the conceptual model proposed by Stodden et al. [2]. According to this model, the acquisition of motor skills during early childhood serves as a critical foundational mechanism that drives subsequent engagement in physical activity. A lack of early exercise experiences may hinder motor skill development, ultimately leading to a negative trajectory of physical inactivity [3]. Although physical growth and motor skill development do not always follow parallel trajectories [4], establishing these foundational skills is essential [5]. Furthermore, evidence suggests that the pathways of motor skill development may differ by sex [6].

Previous studies indicate that motor skills are positively associated with physical activity in early childhood [7,8]. For example, jumping movements, including double-leg continuous jumps, require not only lower-body strength but also coordination skills such as rhythm and timing [9]. In addition, the relationship between motor skills and physical activity may differ between boys and girls [10]. In Japan, increasing polarization in participation in physical activity has been observed, particularly among girls [11,12].

These findings suggest that even in early childhood, differences in early exercise experiences may exist by sex; however, longitudinal studies examining motor skill development while considering age, sex, and types of motor skills remain limited [13,14]. Furthermore, differences in biological maturation rates and differential environmental influences, such as societal expectations regarding physical play, may further contribute to the distinct developmental patterns observed between boys and girls.

Both home and preschool environments are essential for early childhood motor development. Home opportunities, including parental involvement and outdoor play, significantly influence early exercise experiences [15,16]. Similarly, preschools provide structured and unstructured physical activity that supports the development of motor skills [17,18]. While both forms of activity promote skill acquisition, autonomy, and enjoyment, research suggests that preschool physical activity levels are often insufficient [19,20].

Since early exercise experiences in the home environment may be associated with the development of motor skills in the preschool years, understanding young children’s motor development may require consideration of both home and preschool environments. However, few longitudinal studies have examined how early home-based exercise experiences influence multiple motor skills across age and sex, particularly within the Japanese context, highlighting a need for further investigation.

While ‘Physical Activity’ generally refers to any bodily movement [21], in this study, ‘Early Exercise Experiences’ are specifically defined as the frequency of physical play and active movement opportunities in the home environment. Meanwhile, ‘Motor Skills’ represent the underlying capacity to perform diverse motor tasks [22]. Therefore, the purpose of this study was to examine how the level of early exercise experiences at home during early childhood is associated with children’s motor skills, categorized by age group, type of motor skill, and sex. We hypothesized that children with abundant early exercise experiences would demonstrate superior motor skills performance compared to those with limited experiences, and that these associations would vary by sex and motor skill type, potentially showing a catch-up effect in motor development over time.

To achieve this objective, this study integrated three-year longitudinal follow-up data with parent questionnaire data, analyzing annual motor skill measurements conducted at preschool alongside early exercise experiences at home at the individual level. Research linking early childhood motor skills with the home environment using longitudinal data remains limited. Therefore, this study provides an opportunity to further examine the trajectories of motor skill development, including potential sex-specific differences.

## 2. Materials and Methods

### 2.1. Study Participants

It is important to note that the participants were recruited from a single private preschool, which inherently limits the external validity and generalizability of our findings to broader populations. The participants in this study initially comprised 262 children enrolled in a private preschool in City A for whom complete longitudinal follow-up data across three years (ages 3 to 5) were available. The overall data collection period spanned from 2020 to 2025, encompassing multiple cohorts of children who entered the preschool during this timeframe (e.g., those who entered at age 3 in 2020 and graduated at age 5 in 2022, through to those who entered in 2023 and completed their program in 2025).Of these, 159 children (75 boys, 84 girls) were included in the final analysis because parental questionnaire responses were available and could be linked to the motor skills data at the individual level; children without corresponding parental data were excluded. Because the parent questionnaire regarding early home environment was newly introduced for the cohort entering in 2020, a subset of these participants (*n* = 39) overlaps with our previous research which analyzed historical trends in motor skills from 2016 to 2020 without questionnaire data [23].

The characteristics of the study participants are presented in Table 1. To assess potential selection bias arising from this exclusion, we compared baseline motor scores and physical characteristics measured at age 3 between the included (*n* = 159) and excluded (*n* = 103) children. No significant differences were observed in any metrics (all *p* > 0.05), supporting the representativeness of our final sample (Table 2). Additionally, preliminary analyses using Welch’s *t*-tests confirmed that there were no significant differences in baseline physical characteristics between the abundant and limited exercise experience groups for either boys or girls (height: *p* = 0.589 for boys, *p* = 0.730 for girls; weight: *p* = 0.702 for boys, *p* = 0.934 for girls). However, as expected, the abundant group already exhibited significantly better performance in specific motor skills at baseline (specifically, double-leg continuous jump for boys, *p* = 0.025; plank duration for boys, *p* = 0.004, and girls, *p* = 0.002), reflecting their greater early childhood exercise experiences.

Written informed consent was obtained from preschool principals, childcare provid ers, and parents after the purpose, significance, and procedures of the research were explained. Due to the young age of the participants (3 to 5 years old), formal verbal assent was not obtained from the children; however, they were free to opt out of the activities at any time without any disadvantage. This longitudinal study was conducted in accordance with the Declaration of Helsinki, and approved by the Education and Research Support Center of Osaka Seikei Educational Institution (Initial Approval No. College 2022-13; Continued Approval No. University 2023-78).

### 2.2. Survey Method

We obtained the longitudinal results of the five motor skill tests administered at the preschool for children enrolled from 2020 to 2025. Parent surveys were conducted using Google Forms for parents of children enrolled in the younger (3-year-old), middle (4-year-old), and older (5-year-old) classes in December 2022, as well as for parents of children enrolled in the younger class in January 2024, achieving a response rate of 65%. In these surveys, parents were asked to retrospectively report on their children’s early active play and exercise experiences prior to preschool enrollment (up to approximately age 3). Although this questionnaire was designed to capture a broad overview of early activity patterns, we acknowledge the absence of formal psychometric validation, and therefore, it should be interpreted as a parent-reported subjective indicator.

### 2.3. Motor Skill Assessments

Motor skill assessments for preschoolers were conducted annually in October. The motor ability test items utilized in this study are widely adopted in Japan to objectively evaluate early childhood motor competence. According to the Early Childhood Physical Activity Guidelines Handbook [24], “In early childhood, motor ability develops through experiencing diverse movements. Implementing this relatively simple test battery allows for an objective assessment of young children’s motor ability based on fundamental movements such as running, jumping, and throwing.”

The assessments included five items: a 25 m dash, standing long jump, tennis ball throw, double-leg continuous jumps, and plank duration. Time-based measurements (25 m dash, double-leg continuous jumps, and plank duration) were recorded using digital stopwatches (CASIO Stopwatch HS-3C-8AJH, Casio Computer Co., Ltd., Tokyo, Japan). Distance-based measurements (standing long jump and tennis ball throw) were recorded using glass fiber tape measures (Hayamaki 12 double-sided 12-30HRW, Sekisui Resin Co., Ltd., Osaka, Japan). The validity and reliability of these standardized measurement procedures and the overall test battery for evaluating early childhood physical competence have been well-established in the previous literature [24,25].

Among these items, plank duration—which corresponds to “body support duration” in the Japanese standard [24]—was measured using a specific protocol: platforms were placed on both sides of the child’s body at elbow height and shoulder width. The child placed their hands on the platforms and, upon a signal, extended their arms to lift their feet off the floor. The duration was recorded until the child could no longer support their body weight with both arms, with a maximum duration of 180 s. While this item specifically reflects core muscular endurance and stability rather than a fundamental movement skill itself, it is included here because it is traditionally integrated into the Japanese standardized test battery as a key component of overall physical competence.

Each assessment was strictly administered by childcare providers and trained student assistants following the established guidelines [24] to ensure measurement consistency. To evaluate the longitudinal reliability of the motor skill test battery, Intraclass Correlation Coefficients (ICCs) were calculated for each test across the three time points (ages 3, 4, and 5) using a two-way mixed model (consistency). Across the entire sample, the single-measure ICCs ranged 0.242–0.490, which appropriately reflects the natural developmental variability and individual rank-order changes characteristic of this rapid growth period in early childhood.

### 2.4. Parent Questionnaire

The parent survey was developed with reference to Survey 3-1: Parent Interviews from the “Research on Practical Activities in Early Childhood to Cultivate the Foundations for Improving Physical Fitness” [26]. To assess parents’ awareness and children’s physical activity experiences prior to preschool enrollment, items related to early active play and exercise experiences and parental perceptions were selected. In this study, responses to Question 10—“Did your child engage in a lot of physical play or exercise by around age 3?”—were analyzed. Based on this dichotomous question, participants were classified into two categories regarding their ‘Early Exercise Experiences’: ‘Abundant’ (Yes) and ‘Limited’ (No). These labels were chosen to represent the caregivers’ perceived relative frequency and richness of their child’s early-life physical activity. While ‘Abundant’ reflects a high level of engagement in diverse physical play and exercise, ‘Limited’ indicates a lower level of such engagement, as perceived by the parents.

### 2.5. Statistical Analysis

Based on responses to Question 10 of the parent questionnaire, participants were classified into two groups: (1) children with abundant exercise experiences prior to preschool enrollment (“Yes”) and (2) children with limited exercise experiences (“No”). As previously described, the exercise experiences referred to the period prior to preschool enrollment, whereas motor skills were measured longitudinally over the three years of preschool. A two-factor mixed-design analysis of variance (ANOVA) was conducted separately for boys and girls to examine the developmental trajectories of physical growth (height and weight) and motor skills. Exercise experience (two levels: abundant vs. limited) served as the between-subjects factor, and age (three levels: ages 3, 4, and 5) served as the within-subjects repeated measure. Prior to the ANOVAs, statistical assumptions were evaluated. Normality was assessed using the Shapiro–Wilk test; although some variables (e.g., plank duration and double-leg continuous jumps) showed deviations from normality (Statistic range: 0.535–0.994; *p* < 0.05 for non-normal variables, see Supplementary Table S1), ANOVA was utilized due to its general robustness. To confirm that our findings were not artifacts of distributional violations, we performed rank-transformed sensitivity analyses (Supplementary Table S2). While these sensitivity analyses generally supported our primary ANOVA results, two items (25 m dash and double-leg continuous jump) showed attenuated significance; thus, these specific findings should be interpreted with caution. The assumption of sphericity was assessed using Mauchly’s test, and the Greenhouse-Geisser correction was applied when this assumption was violated. Furthermore, to address potential cohort-level confounding due to the COVID-19 pandemic, we performed sensitivity analyses by including the enrollment cohort as a between-subjects factor in our longitudinal models (Supplementary Table S3). Finally, to explore whether the influence of early exercise experiences on motor development differed by sex, we conducted a three-way ANOVA (Exercise Experience × Age × Sex) and reported the interaction effects (Supplementary Table S4). Given the retrospective grouping, an a priori power analysis was not possible, leading to an unequal sample size distribution (e.g., limited experience boys, *n* = 19). Furthermore, to address multiplicity across the 14 ANOVA models, we prioritized the evaluation of effect sizes (partial eta squared, $\eta_{p}^{2}$) and 95% *CI* rather than strict alpha-level adjustments, which could over-inflate Type II errors in this exploratory setting. When significant interactions were observed, simple main effects tests were conducted. All analyses were performed using IBM SPSS Statistics version 30 (IBM Corp., Armonk, NY, USA), with the significance level set at *p* < 0.05. Note that the limited sample sizes in the ‘Limited’ experience groups (boys: *n* = 19; girls: *n* = 25) may result in restricted statistical power; thus, findings—particularly those with borderline significance (e.g., *p* = 0.04)—should be interpreted with caution.

## 3. Results

This study conducted a two-factor mixed-design analysis of variance (ANOVA) on preschool children’s physical growth (height and weight) and five motor skills, with age (ages 3, 4, and 5) as the within-subjects factor and early exercise experiences (abundant vs. limited) as the between-subjects factor, analyzed separately by sex. Means and standard deviations for physical growth (height and weight) and motor skills by sex are presented in Table 3. The results of the mixed-design ANOVA, including the main effects of age and exercise experiences, as well as their interactions, are provided in Table 4.

For boys, the abundant exercise experience group showed significantly better performance than the limited experience group across multiple motor skills. Specifically, significant main effects of exercise experience were observed for the 25 m dash (mean difference = −0.58 s; 95% CI [−1.07, −0.10]; *p* = 0.020), standing long jump (mean difference = 10.23 cm; 95% CI [1.50, 18.96]; *p* = 0.022), and plank duration (mean difference = 16.42 s; 95% CI [5.07, 27.77]; *p* = 0.005). Regarding the double-leg continuous jump, a significant age × exercise experience interaction was observed (*F* = 3.335; *p* = 0.041; $\eta_{p}^{2}$ = 0.044). Simple main effects analysis revealed that the abundant exercise group significantly outperformed the limited group at age 3 (mean difference = −1.037 s; 95% CI [−1.824, −0.251]; *p* = 0.010). In contrast, no significant differences were observed between the two experience groups for girls across any of the motor skills measured (all *p* > 0.05). To formally examine whether developmental trajectories differed by gender, a three-way repeated measures ANOVA (Age × Exercise Experience × Gender) was conducted for all five motor skill tasks. The three-way interaction effects for all motor skills did not reach statistical significance: 25 m dash (*F* = 2.814, *p* = 0.095), standing long jump (*F* = 2.133, *p* = 0.146), tennis ball throw (*F* = 0.816, *p* = 0.368), double-leg continuous jump (*F* = 1.866, *p* = 0.174), and plank duration (*F* = 2.607, *p* = 0.108). These results suggest that the influence of early exercise experiences on developmental trajectories does not significantly vary by gender for these motor tasks.

### 3.1. Physical Growth (Height and Weight)

For both boys and girls, the main effect of age on height and weight was significant, with both measures predictably increasing as age advanced (*p* < 0.001). In contrast, neither the main effect of early exercise experiences nor the interaction between early exercise experiences and age reached significance (*p* > 0.05). Therefore, no significant differences in physical growth (height and weight) were observed between the abundant and limited exercise experience groups in either sex.

### 3.2. Motor Skills (Five Events)

**Figure 1:**
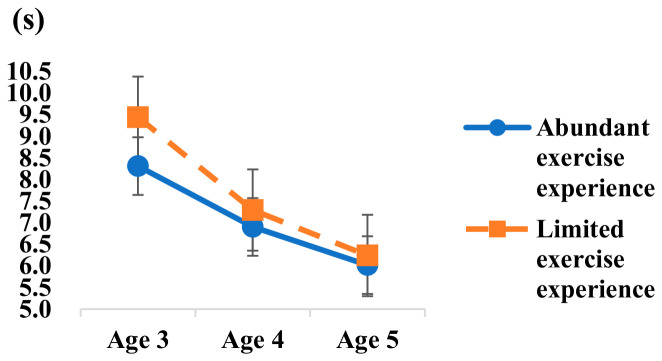
Developmental changes in 25 m dash among boys by early exercise experiences. Note: Error bars represent 95% confidence intervals (95% CI).

**Figure 2:**
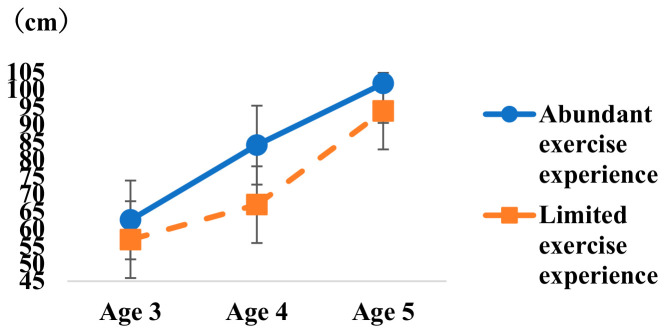
Developmental changes in standing long jump among boys by early exercise experiences. Note: Error bars represent 95% confidence intervals (95% CI).

**Figure 3:**
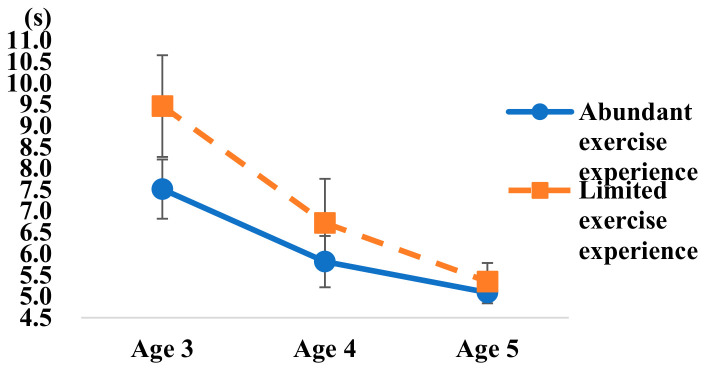
Developmental changes in double-leg continuous jump among boys by early exercise experiences. Note: Error bars represent 95% confidence intervals (95% CI).

**Figure 4:**
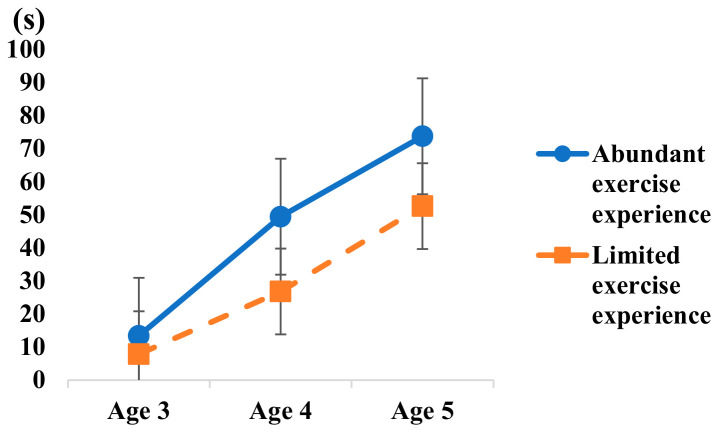
Developmental changes in plank duration among boys by early exercise experiences. Note: Error bars represent 95% confidence intervals (95% CI).

Among boys, the main effect of early exercise experiences was significant for four of the five motor skills: the 25 m dash (Figure 1), standing long jump (Figure 2), double-leg continuous jump (Figure 3), and plank duration (Figure 4), with the abundant experience group consistently demonstrating superior performance (Table 4). The tennis ball throw was the only exception. Regarding developmental trajectories, a significant age × exercise experience interaction was observed specifically for the double-leg continuous jump among boys (*F*[2, 146] = 3.335, *p* = 0.041, $\eta_{p}^{2}$ = 0.044; Figure 3). Although this effect size is relatively small, it nonetheless indicates that the developmental trajectories of this motor skill differed depending on the abundance of early exercise experiences.

**Figure 5:**
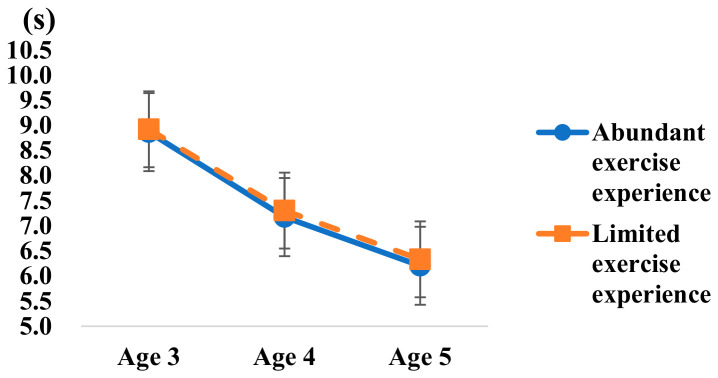
Developmental changes in 25 m dash among girls by early exercise experiences. Note: Error bars represent 95% confidence intervals (95% CI).

**Figure 6:**
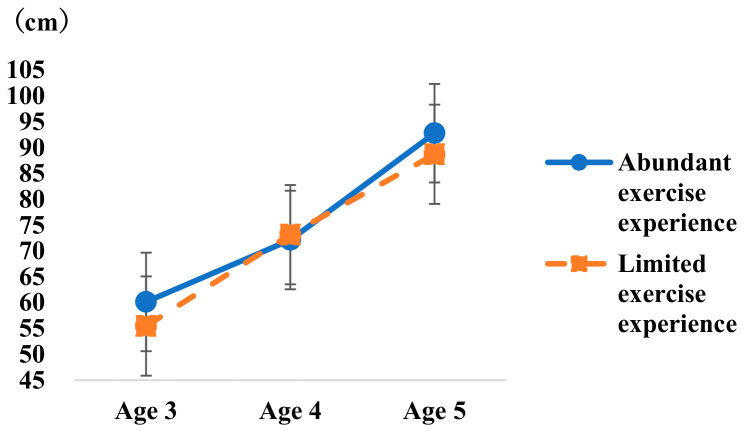
Developmental changes in standing long jump among girls by early exercise experiences. Note: Error bars represent 95% confidence intervals (95% CI).

**Figure 7:**
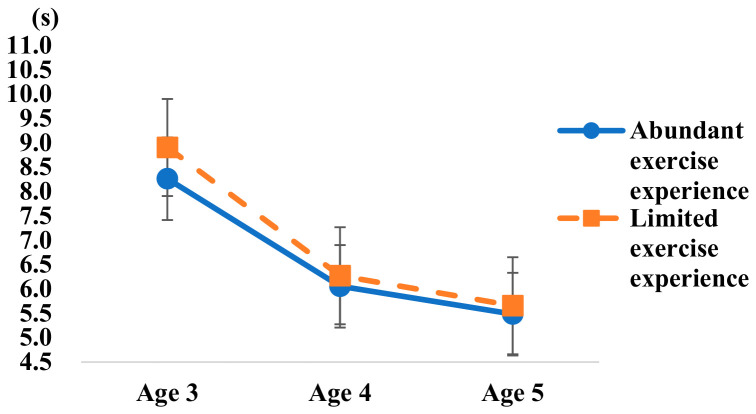
Developmental changes in double-leg continuous jump among girls by early exercise experiences. Note: Error bars represent 95% confidence intervals (95% CI).

**Figure 8:**
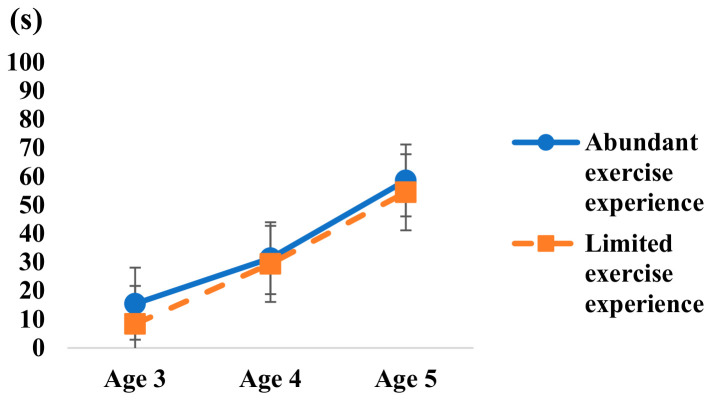
Developmental changes in plank duration among girls by early exercise experiences. Note: Error bars represent 95% confidence intervals (95% CI).

In contrast, among girls, neither the main effect of early exercise experiences nor its interaction with age reached significance for any of the measured skills (Figure 5, Figure 6, Figure 7 and Figure 8). Regarding the potential influence of sex on developmental trajectories, a three-way repeated measures ANOVA (Age × Exercise Experience × Gender) revealed no statistically significant three-way interaction effects for any of the five motor skills (Supplementary Table S4). This indicates that the influence of early exercise experiences on motor development did not significantly differ by gender; thus, reporting separate results for boys and girls reflects a descriptive approach rather than implying a statistically significant sexual dimorphism in the effect of early experience. Finally, the developmental trajectories for the four motor skills (excluding the tennis ball throw, for which no significant effects were observed in either boys or girls) are illustrated in Figure 1, Figure 2, Figure 3, Figure 4, Figure 5, Figure 6, Figure 7 and Figure 8. Although the effect sizes ($\eta_{p}^{2})$ were modest, these findings represent meaningful developmental gaps during the critical period of early childhood.

## 4. Discussion

In this study, motor skills were treated as measurable outcomes, distinct from the broader concept of physical activity. The use of longitudinal data linked with parent-reported early exercise experiences provides a valuable perspective for understanding developmental changes in motor skills during early childhood.

The results showed that, among boys, differences in certain motor skills were significantly associated with early home-based exercise experiences, whereas no consistent differences were observed among girls. Regarding physical growth (height and weight), no significant differences were found between the abundant and limited exercise experience groups in either sex. Although prior research has reported associations between physical growth and motor skills during early childhood [27], the magnitude and direction of these associations remain complex and inconsistent across studies [28].

Because our study found no significant group differences in physical growth (height and weight), the results suggest that the superior motor performance observed in boys with abundant early exercise experiences is likely associated with the experience itself rather than being primarily attributable to differences in physical growth. Furthermore, the findings of this study are consistent with previous research indicating that the influence of home environments on motor skill development may vary by sex [29].

Regarding the specific types of movements, boys in the abundant group demonstrated superior performance in four of the five motor skills, with the tennis ball throw being the only exception. This indicates that early exercise experiences are not uniformly associated with all motor skills, suggesting that factors influencing motor skill development may vary depending on the specific type of skill [30].

Among boys, differences between the abundant and limited groups exhibited different longitudinal patterns depending on the type of motor skill. More persistent differences were observed in skills relying heavily on muscle strength, such as plank duration. These patterns suggest that the benefits of abundant early exercise experiences may persist for strength-based tasks. In contrast, physical activity within preschool settings may have the potential to contribute to the development of running speed and agility, thereby allowing children with limited early experiences to potentially catch up over time. The fact that these early differences and subsequent catch-up patterns were prominent in boys, but absent in girls, may reflect gender differences in activity preferences and socialization processes during free play at preschool [31,32]. Broadly, these findings can be interpreted within an experience-dependent framework, in which early physical activity provides initial opportunities for repeated engagement with movement, supporting the foundational development of motor skills.

Specifically, the significant interaction observed in the double-leg continuous jump provides preliminary evidence for a potential catch-up effect. This motor skill requires not only lower-body strength but also complex coordination skills, including rhythm and timing [9]. Notably, the observed interaction indicates that initial performance gaps at age 3 associated with limited early exercise experiences appeared to diminish over time during the preschool years. Initial differences between the abundant and limited groups at age 3 (a mean difference of 1.95 s) decreased over time, with the performance gap showing a substantial reduction by age 5 (a mean difference of 0.26 s). This pattern highlights that the effect of early exercise experiences is not uniform across all motor skills; rather, for coordination-heavy skills, standard preschool environments might provide sufficient stimuli to mitigate the developmental gap by age 5 [33].

While these findings suggest a catch-up effect in motor competence, this interpretation warrants cautious consideration alongside several alternative mechanisms. First, natural maturation may play a significant role, as the rapid physiological development during the preschool years could naturally diminish initial performance disparities. Second, the standard preschool curriculum likely serves as a critical equalizer, providing structured physical activity that mitigates the initial disadvantage of limited home-based exercise. Finally, the phenomenon of regression toward the mean must be acknowledged; children who initially exhibited extreme scores may naturally move toward the group average in subsequent assessments. Crucially, these factors are not mutually exclusive; rather, they likely operate in tandem with the positive influence of early exercise experiences, suggesting that the developmental trajectory of motor skills is a multifaceted process shaped by both individual history and the preschool environment.

Previous research has noted that the development of coordination-based motor skills during early childhood does not necessarily correlate strongly with physical growth (height and weight) indicators [34], and the results of this study align with these skill-specific characteristics. Specifically, no significant differences were observed in the tennis ball throw based on early exercise experiences. Throwing involves multiple technical components, including coordination among different body segments and the acquisition of complex movement patterns. Prior studies have suggested that successful development in throwing depends not only on the amount of exercise experience but also on its content, quality, and direct instructional guidance [35]. While direct data on specific throwing opportunities were not captured in this study—making this interpretation speculative—it is plausible to hypothesize that the lack of group differences in this specific skill may reflect generally limited opportunities for targeted throwing activities among all children in this sample, regardless of their overall exercise abundance. Future research employing detailed activity logs or direct observation is necessary to validate this possibility.

Regarding the female cohort, no consistent differences in motor skills were observed between the abundant and limited early exercise experience groups. This suggests that girls’ motor skills may be influenced more heavily by factors outside the home environment, such as the design of activities in childcare settings, opportunities for physical activity in the local community, and broader sociocultural factors [36]. Our findings suggest that developmental trajectories in motor competence can be influenced by both environmental stimuli and individual factors, including gender. Recent studies have highlighted how restrictive or intervention-based environments can differentially impact children’s health outcomes. Specifically, research during the COVID-19 pandemic revealed that girls and boys exhibit distinct psychological and physical responses to environmental changes [37]. Furthermore, integrated approaches—such as combining structured physical activity with neuro-stimulation—have been shown to enhance functional outcomes by addressing individual needs through neuroplasticity [38]. These insights support our observed sex-specific patterns, reinforcing the notion that the developmental benefits of physical activity are multifaceted and mediated by gender-dependent factors.

Consequently, understanding motor development in girls requires a multifaceted approach that considers not only quantitative aspects—such as the abundance of early exercise experiences—but also the qualitative nature of physical play and the surrounding environment [39]. While our study did not directly measure the specific opportunities for physical activity within preschool settings—meaning that our conclusions regarding these environmental influences remain speculative—it is reasonable to propose that environmental factors—particularly the selection and quality of physical play in preschool settings— likely serve as crucial drivers of motor skill development for girls, highlighting the vital compensatory role of early childhood education environments.

### 4.1. Practical Implications

These findings can be effectively interpreted within an ecological systems perspective, suggesting that motor development is holistically shaped through dynamic interactions between home and preschool environments. Given the observational nature and the modest effect sizes observed in this study, our findings offer preliminary insights rather than definitive guidelines for preschool educators. Because the relationship between early motor skill development and the home environment varies depending on both sex and the specific type of motor skill, it is crucial to consider diverse forms of physical activity across developmental stages, rather than focusing solely on the abundance of early exercise experiences. Consequently, educators may consider designing curricula that prioritize rhythmic and coordination-based activities, which appear particularly promising for developmental catch-up. While further research is required to confirm these interventions, our study underscores the potential value of structured physical activity in creating an inclusive developmental environment for all children.

### 4.2. Limitations and Future Directions

This study has several limitations. First, because the sample was drawn from a single private preschool, caution is needed when generalizing the results, and it implies potential clustering that was not modeled in the analysis. Additionally, the data collection period overlapped with the COVID-19 pandemic, which may have uniquely influenced children’s physical activity opportunities, and the 65% parental questionnaire response rate introduces potential selection bias. To address this, we compared baseline motor performance between responders and non-responders and found no significant differences, suggesting that the impact of non-response bias on our findings may be limited. Second, early exercise experiences were captured as a binary variable (abundant vs. limited) based on a single retrospective parental self-report, which introduces recall bias and potential exposure misclassification, and may not fully reflect the exact frequency, content, or qualitative aspects of such activity. Furthermore, we acknowledge that the reliability and validity of our single-item parental questionnaire were not formally assessed. This limitation, combined with the retrospective nature of the reporting, implies that our measure of ‘early exercise experience’ should be interpreted as a subjective indicator rather than a precise quantification of activity. In addition the study lacks objective physical activity assessments. Third, the relatively small number of boys in the limited exercise experience group (n = 19) may reduce statistical precision. Fourth, we did not control for potential confounding factors, including family socioeconomic status, parental educational background, home environment, and genetic predisposition or possible differences in extracurricular activities outside the preschool. Fifth, while we emphasize the potential compensatory role of the preschool environment in bridging early developmental gaps, it is important to note that our study did not directly measure the specific physical activity content or quality provided by the preschool curriculum. Consequently, our interpretation regarding the role of the preschool environment is based on the observed narrowing of performance gaps across age groups, which warrants further verification through direct assessment of preschool-based physical activity programs. Furthermore, the reduction in performance differences between the two groups over time should be interpreted with caution. While we hypothesize a compensatory effect of the preschool environment, we acknowledge that other factors—such as natural developmental maturation, regression to the mean, or varied participation levels in other extracurricular activities—may also contribute to this convergence. Therefore, our study serves to suggest a potential role for preschool education rather than providing definitive proof of its compensatory mechanism.

Future studies using objective physical activity measurements and longitudinal designs that incorporate socioeconomic and extracurricular data are warranted to confirm these findings. Consequently, definitive causal conclusions cannot be drawn, and alternative explanations for the observed catch-up effect, such as natural maturation or regression to the mean, cannot be ruled out. Furthermore, due to the exploratory nature of the analyses across multiple motor skills, the findings regarding specific group differences should be interpreted with awareness of potential multiplicity. Despite these limitations, this study provides valuable insight into the longitudinal relationship between early exercise experiences and subsequent motor skills, analyzed by age group, sex, and type of motor skill. Finally, given the exploratory nature of this study, we did not apply stringent multiple comparison corrections (e.g., Bonferroni correction). While this increases the risk of Type I errors, we aimed to avoid overly conservative thresholds that might obscure preliminary evidence in this under-researched area. However, it should be noted that applying the Benjamini–Hochberg correction for multiple testing (e.g., across 14 tests for exercise experiences and age-by-exercise interactions in boys) reveals that no results survive a 5% false discovery rate (FDR) threshold, including the nominal significance for plank duration (*p* = 0.005). Additionally, the minimum detectable effect sizes (partial eta squared) given our sample sizes were approximately 0.097 for boys and 0.087 for girls. Since some of the statistically significant effects reported herein fall below these thresholds, our findings—particularly the age × exercise experience interaction in the double-leg continuous jump—should be interpreted as hypothesis-generating, and they require validation in future studies with larger samples. Despite these limitations, this study provides valuable insight into the longitudinal relationship between early exercise experiences and subsequent motor skills, analyzed by age group, sex, and type of motor skill.

## 5. Conclusions

The results of this study indicate that early exercise experiences were significantly associated with certain motor skills in boys; however, these associations were not uniform across specific motor skills or age groups. In contrast, no consistent differences were observed among girls based on the abundance of early exercise experiences. These findings highlight that early motor skill development must be understood by considering multiple factors. Ultimately, these findings highlight that comprehensive environmental support may be beneficial, as both home and early childhood education settings likely play complementary roles in fostering motor development during early childhood. However, as preschool environmental characteristics were not directly measured, these interpretations should be viewed as plausible hypotheses rather than definitive conclusions. Given the exploratory nature of these associations, future research using larger, multi-center, prospective longitudinal studies is required to validate these findings and further elucidate the mechanisms of motor skill development.

## Figures and Tables

**Table 1:** Participant Characteristics and Physical Stature by Sex.

	Boys (*n* = 75)		Girls (*n* = 84)	
Item	Mean	SD	Mean	SD
Height (Age 3), cm	100.26	4.77	98.42	4.45
Height (Age 4), cm	106.85	4.97	105.22	4.71
Height (Age 5), cm	113.31	5.18	111.56	5.06
Weight (Age 3), kg	15.34	1.69	14.83	1.80
Weight (Age 4), kg	17.11	2.08	16.68	2.12
Weight (Age 5), kg	19.15	2.52	18.75	2.80
Abundant Exercise Experience, *n*	56		59	
Limited Exercise Experience, *n*	19		25	

Note. SD = standard deviation. Height and weight at ages 3, 4, and 5 were measured longitudinally in the same children over a three-year period.

**Table 2:** Comparison of baseline characteristics at age 3 between included and excluded children.

Measurement Items	Boys		Boys			Girls		Girls		
	(Included, *n* = 75)		(Excluded, *n* = 50)			(Included, *n* = 84)		(Excluded, *n* = 53)		
	Mean	SD	Mean	SD	*p*	Mean	SD	Mean	SD	*p*
Height (cm)	100.26	4.77	100.07	3.80	0.807	98.42	4.45	99.45	4.30	0.179
Weight (kg)	15.34	1.69	15.28	1.67	0.835	14.83	1.80	15.04	1.78	0.518
25 m dash (s)	8.60	1.89	8.77	1.54	0.575	8.88	1.27	9.06	1.42	0.477
Standing long jump (cm)	61.17	17.59	58.26	18.28	0.378	58.76	16.59	56.09	18.51	0.395
Tennis ball throw (m)	3.58	1.54	3.45	1.45	0.634	2.88	0.93	2.64	0.95	0.162
Double-leg continuous jump (s)	8.01	2.73	8.73	3.22	0.197	8.45	3.11	9.89	5.53	0.088
Plank duration (s)	11.98	9.02	11.24	8.69	0.647	13.38	13.59	10.26	8.66	0.104

Note. SD = standard deviation. All *p*-values represent the results of independent *t*-tests comparing baseline characteristics at age 3 between included and excluded children.

**Table 3:** Means and standard deviations for physical stature and motor skills by sex and early exercise experiences.

Measurement Items		Boys		Boys		Girls		Girls	
		(Abundant, *n* =56)		(Limited, *n* = 19)		(Abundant, *n* = 59)		(Limited, *n* = 25)	
	Age	Mean	SD	Mean	SD	Mean	SD	Mean	SD
Height (cm)	3	100.41	5.12	99.82	3.62	98.53	4.43	98.15	4.55
	4	106.93	5.32	106.59	3.86	105.41	4.67	104.78	4.87
	5	113.37	5.56	113.13	3.95	111.77	5.02	111.07	5.21
Weight (kg)	3	15.30	1.70	15.47	1.69	14.82	1.89	14.86	1.62
	4	17.10	2.13	17.15	1.96	16.79	2.32	16.40	1.55
	5	19.06	2.58	19.40	2.42	18.78	3.00	18.68	2.29
25 m dash (s)	3	8.31	1.32	9.44	2.88	8.87	1.30	8.92	1.20
	4	6.91	0.69	7.29	0.95	7.17	0.61	7.30	0.61
	5	6.02	0.94	6.24	0.71	6.21	0.49	6.33	0.58
Standing long jump (cm)	3	62.61	16.46	56.95	20.45	60.15	16.02	55.48	17.76
	4	84.11	19.76	67.00	21.29	72.14	14.56	73.16	17.93
	5	101.82	23.09	93.89	22.32	92.80	14.39	88.72	20.04
Tennis ball throw (m)	3	3.68	1.41	3.29	1.91	2.90	0.89	2.82	1.03
	4	5.73	2.21	4.37	1.23	4.28	1.20	3.70	1.10
	5	7.66	2.81	6.76	3.10	5.56	1.93	4.94	1.56
Double-leg continuous jump (s)	3	7.51	2.35	9.46	3.27	8.26	2.99	8.90	3.39
	4	5.81	1.92	6.72	3.08	6.05	1.37	6.27	1.07
	5	5.09	0.92	5.35	1.06	5.48	0.94	5.66	0.87
Plank duration (s)	3	13.38	9.54	7.84	5.72	15.48	15.44	8.41	5.10
	4	49.39	33.00	26.78	17.08	31.38	21.00	29.40	24.09
	5	73.70	37.65	52.58	37.73	58.59	37.69	54.44	34.59

Note. SD = standard deviation.

**Table 4:** Results of two-factor mixed-design analysis of variance (age × early exercise experience).

		Boys (*n* = 75)			Girls (*n* = 84)		
Measurement Items	Factor	*F*	*p*	$\eta_{p}^{2}$	*F*	*p*	$\eta_{p}^{2}$
Height (cm)	Age	5878.621	<0.001 ***	0.988	5856.550	<0.001 ***	0.986
	Exercise experience	0.087	ns	0.001	0.256	ns	0.003
	Age × exercise experience	1.083	ns	0.015	0.996	ns	0.012
Weight (kg)	Age	330.372	<0.001 ***	0.819	389.111	<0.001 ***	0.826
	Exercise experience	0.123	ns	0.002	0.083	ns	0.001
	Age × exercise experience	0.456	ns	0.006	1.192	ns	0.014
25 m dash (s)	Age	99.013	<0.001 ***	0.576	263.133	<0.001 ***	0.762
	Exercise experience	5.674	0.020 *	0.072	0.436	ns	0.005
	Age × exercise experience	2.977	ns	0.039	0.062	ns	0.001
Standing long jump (cm)	Age	98.723	<0.001 ***	0.575	133.720	<0.001 ***	0.620
	Exercise experience	5.454	0.022 *	0.070	0.704	ns	0.009
	Age × exercise experience	2.477	ns	0.033	1.204	ns	0.014
Tennis ball throw (m)	Age	72.532	<0.001 ***	0.498	83.143	<0.001 ***	0.503
	Exercise experience	3.580	ns	0.047	3.017	ns	0.035
	Age × exercise experience	1.230	ns	0.017	1.322	ns	0.016
Double-leg continuous jump (s)	Age	50.997	<0.001 ***	0.411	53.957	<0.001 ***	0.397
	Exercise experience	6.911	0.010 *	0.086	1.120	ns	0.013
	Age × exercise experience	3.335	0.041 *	0.044	0.353	ns	0.004
Plank duration (s)	Age	77.742	<0.001 ***	0.516	90.844	<0.001 ***	0.526
	Exercise experience	8.313	0.005 **	0.102	0.824	ns	0.010
	Age × exercise experience	2.515	ns	0.033	0.296	ns	0.004

* *p* < 0.05, ** *p* < 0.01, *** *p* < 0.001, ns = non-significant.

## Data Availability

The data presented in this study are available on request from the corresponding author. The data are not publicly available due to privacy restrictions.

## Supplementary Materials

The following supporting information can be downloaded at https://www.mdpi.com/article/10.3390/children13070947/s1, Table S1: Results of the Shapiro-Wilk Test for Normality.; Table S2: Sensitivity Analysis of the Primary ANOVA Results Using Rank-Transformed Data.; Table S3: Impact of Enrollment Cohort on Motor Performance (COVID-19 Cohort Consistency Check).; Table S4: Interaction Effects Between Gender and Early Exercise Experience on Motor Performance (Three-Way ANOVA).

## References

[1] Sports Agency of Japan (2025). Summary of the Results of the FY2025 National Survey of Physical Fitness, Athletic Performance, and Exercise Habits.

[2] Stodden D.F., Goodway J.D., Langendorfer S.J., Roberton M.A., Rudisill M.E., Garcia C., Garcia L.E. (2008). A Developmental Perspective on the Role of Motor Skill Competence in Physical Activity: An Emergent Relationship. Quest.

[3] Otsubo K., Kasuga K., Yamatsugi S., Nakano T. (2024). Relationship between physical fitness and academic ability of elementary school children and habits that influence them as seen from a multifaceted analysis. Jpn. J. Phys. Educ. Health Sport Sci..

[4] Kojić F., Pelemiš V., Jorgić B., Olanescu M., Suciu A., Peris M. (2023). Relationship between body composition and gross motor coordination in six-year-old boys and girls. Appl. Sci..

[5] Adolph K.E., Franchak J.M. (2017). The development of motor behavior. Wiley Interdiscip. Rev. Cogn. Sci..

[6] Oikawa N. (2018). Effects of birth physique and motor development in infancy on fundamental motor abilities in early childhood. Jpn. J. Child Health.

[7] Jones D., Innerd A., Giles E.L., Azevedo L.B. (2020). Association between fundamental motor skills and physical activity in the early years: A systematic review and meta-analysis. J. Sport Health Sci..

[8] Xin F., Chen S.T., Clark C., Hong J.T., Liu Y., Cai Y.J. (2020). Relationship between fundamental movement skills and physical activity in preschool-aged children: A systematic review. Int. J. Environ. Res. Public Health.

[9] Takatoku N. (2019). The effect of two-footed synchronism on spatiotemporal body control while hopping in young children. Taiikugaku Kenkyu.

[10] Logan S.W., Webster E.K., Getchell N., Pfeiffer K.A., Robinson L.E. (2018). Relationship between motor competence and physical activity. Sports Med..

[11] Kasuga K., Nagano T., Oguri K. (2010). Time of polarization at the physical fitness level in children—Based on the follow-up survey to the past of the group that exists in two poles at five-years old time. J. Educ. Health Sci..

[12] Sports Agency of Japan (2018). Measures to Promote Women’s Sports.

[13] Ogura Y., Kasuga K., Nakano T. (2020). The effect of motor ability development in early childhood on that in 6th grade—Based on the longitudinal data of motor ability test. Jpn. J. Sport Health Sci..

[14] Koolwijk P., de Jonge E., Mombarg R., Remmers T., Van Kann D., van Aart I., de Vries S. (2024). Changes in motor competence of 4–8-Year-Old children: A longitudinal Study. Int. J. Environ. Res. Public Health.

[15] Hinkley T., Crawford D., Salmon J., Okely A.D., Hesketh K. (2008). Preschool children and physical activity: A review of correlates. Am. J. Prev. Med..

[16] Huang T., Zhao G., Fu J., Sun S., Lv W., He Z., Chen R. (2024). Associations between family factors and physical activity clustering in preschool children: A cross-sectional study. Front. Public Health.

[17] Timmons B.W., Naylor P.J., Pfeiffer K.A. (2012). Physical activity for preschool children—How much and how?. Appl. Physiol. Nutr. Metab..

[18] Uchida T., Ooi T., Tsutsui S. (2018). The impact of ladder exercise play, circuit play, and free play in early childhood on improving physical fitness and motor skills: Focus on an exercise program emphasizing intrinsic motivation. Jpn. J. Hum. Growth Dev. Res..

[19] Chen D., Zhao G., Fu J., Shun S., Su L., He Z., Shen F. (2024). Effects of structured and unstructured interventions on fundamental motor skills in preschool children: A metaanalysis. Front. Public Health.

[20] Pate R.R., O’Neill J.R., Brown W.H., Pfeiffer K.A., Dowda M., Addy C.L. (2015). Prevalence of Compliance with a New Physical Activity Guideline for Preschool-Age Children. Child. Obes..

[21] Caspersen C.J., Powell K.E., Christenson G.M. (1985). Physical Activity, Exercise, and Physical Fitness: Definitions and Distinctions for Health-Related Research. Public Health Rep..

[22] Gallahue D.L., Ozmun J.C., Goodway J.D. (2012). Understanding Motor Development: Infants, Children, Adolescents, Adults.

[23] Kobayashi S., Mizukami A. (2024). A Longitudinal Study of Young Children’s Physique and Motor Skills by Year of Entry to Preschool. Jpn. J. Health Educ. Child..

[24] Ministry of Education, Culture, Sports, Science and Technology (2012). Early Childhood Physical Activity Guidelines Guidebook.

[25] Mayhew J.L., Houser J.J., Briney B.B., Williams T.B., Piper F.C., Brechue W.F. (2010). Accuracy of hand-held stopwatches compared with electronic timing in measuring sprint performance. J. Strength Cond. Res..

[26] Ministry of Education, Culture, Sports, Science and Technology (2011). Research Report on the State of Practical Activities in Early Childhood to Cultivate the Foundations for Improving Physical Fitness.

[27] Kakebeeke T.H., Lanzi S., Zysset A.E., Arhab A., Messerli-Bürgy N., Stülb K., Jenni O.G. (2017). Association between body composition and motor performance in preschool children. Obes. Facts.

[28] Rico-González M., Ardigò L.P., Ramírez-Arroyo A.P., Gómez-Carmona C.D. (2024). Anthropometric influence on preschool children’s physical fitness and motor skills: A systematic review. J. Funct. Morphol. Kinesiol..

[29] Zheng Y., Ye W., Korivi M., Liu Y., Hong F. (2022). Gender differences in fundamental motor skills proficiency in children aged 3–6 years: A systematic review and meta-analysis. Int. J. Environ. Res. Public Health.

[30] Escolano-Pérez E., Sánchez-López C.R., Herrero-Nivela M.L. (2021). Early environmental and biological influences on preschool motor skills: Implications for early childhood care and education. Front. Psychol..

[31] Colwell M.J., Lindsey E.W. (2005). Preschool children’s pretend and physical play and sex of play partner: Connections to peer competence. Sex Roles.

[32] Pellegrini A.D., Smith P.K. (1998). Physical activity play: The nature and function of a neglected aspect of play. Child Dev..

[33] Moreira M., Veiga G., Lopes F., Hales D., Luz C., Cordovil R. (2023). Kindergarten affordances for physical activity and preschoolers’ motor and social-emotional competence. Children.

[34] Stupar D., Romanov R., Beretić I. (2025). The relationship between anthropometric measures and manifestation of coordination in preschool children. Anthropologie.

[35] Pratiwi E., Hernawan H., Fachrezzy F., Anggara N., Lestari H., Gumantan A., Samodra Y.T.J., Mappaompo M.A., Juhannis J., Sinulingga A. (2024). Learning model of basic manipulative movements of throwing and catching: A developmental study through early childhood play. Retos.

[36] Biino V., Pesce C., Martins C. (2025). Motor skill development at preschool age in girls and boys: The role of outdoor free play. Children.

[37] Hernández-Pérez H., Cruz-Castruita R.M., Quezada Chacón J., Holguín Ramírez J., Hernández-Cruz G., Alonso-Ramos Z.N., Rangel Colmenero B.R. (2026). Anxiety, depression, and physical activity levels according to sex in children after the COVID-19 lockdown in Mexico. Int. J. Sport Stud. Health.

[38] Akbarimehr H., Ghayour Najafabadi M., Gharayagh Zandi H., Khajavi D. (2026). Effects of aquatic exercise and transcranial direct current stimulation on motor skills and cognitive functions in children with autism spectrum disorder. Int. J. Sport Stud. Health.

[39] Sugihara T., Kawabe T. (2020). Motor Development in Early Childhood and the Teaching of Motor Play: Children Grow Through Play.

