# Supplementary material for: Associations Between Exercise Experiences in the Home Environment During Early Childhood and Motor Skill Development in Preschool Children: A Longitudinal Follow-Up Study
Source: Children (Basel). 2026 Jul 19;13(7):947. doi: 10.3390/children13070947 (PMC13406647; doi:10.3390/children13070947)
Supplement: Supplementary file 1 [file children-13-00947-s001.zip › children-4402289-supplementary.pdf]

TableS1. Results of the Shapiro-Wilk Test for Normality.

		Boys				Girls			
**Measurement Items**	**Age**	**Statistic**	***df***	***P***	**Normality**	**Statistic**	***df***	***P***	**Normality**
Height (cm)	3	0.987	75	0.669	Normal	0.981	84	0.249	Normal
Weight (kg)	3	0.976	75	0.174	Normal	0.983	84	0.343	Normal
25-meter dash (s)	3	0.716	75	0.000	Non-normal	0.939	84	0.001	Non-normal
Standing long jump (cm)	3	0.975	75	0.142	Normal	0.971	84	0.051	Normal
Tennis ball throw (m)	3	0.966	75	0.044	Non-normal	0.936	84	0.000	Non-normal
Double-leg continuous jump (s)	3	0.885	75	0.000	Non-normal	0.862	84	0.000	Non-normal
Plank duration (s)	3	0.904	75	0.000	Non-normal	0.750	84	0.000	Non-normal
Height (cm)	4	0.988	75	0.715	Normal	0.982	84	0.296	Normal
Weight (kg)	4	0.994	75	0.979	Normal	0.954	84	0.004	Non-normal
25-meter dash (s)	4	0.966	75	0.043	Non-normal	0.978	84	0.155	Normal
Standing long jump (cm)	4	0.983	75	0.415	Normal	0.980	84	0.218	Normal
Tennis ball throw (m)	4	0.950	75	0.005	Non-normal	0.962	84	0.015	Non-normal
Double-leg continuous jump (s)	4	0.535	75	0.000	Non-normal	0.910	84	0.000	Non-normal
Plank duration (s)	4	0.890	75	0.000	Non-normal	0.879	84	0.000	Non-normal
Height (cm)	5	0.989	75	0.785	Normal	0.981	84	0.246	Normal
Weight (kg)	5	0.991	75	0.856	Normal	0.927	84	0.000	Non-normal
25-meter dash (s)	5	0.681	75	0.000	Non-normal	0.975	84	0.101	Normal
Standing long jump (cm)	5	0.951	75	0.006	Non-normal	0.988	84	0.617	Normal
Tennis ball throw (m)	5	0.969	75	0.059	Normal	0.935	84	0.000	Non-normal
Double-leg continuous jump (s)	5	0.848	75	0.000	Non-normal	0.847	84	0.000	Non-normal
Plank duration (s)	5	0.958	75	0.015	Non-normal	0.892	84	0.000	Non-normal

TableS2. Sensitivity Analysis of the Primary ANOVA Results Using Rank-Transformed Data.

Measurement Items	Primary ANOVA (*F, P*)	Rank-Transformed ANOVA*(F, P)*
25-meter dash (s)	F = 5.674, p =0.020*	F = 3.895, p = 0.052
Standing long jump (cm)	F = 5.454, p =0.022*	F = 4.797, p =0.032*
Double-leg continuous jump (s)	F = 6.911, p =0.010*<br>Interaction*: F=3.335, p=0.041* *	F = 8.627, p = 0.004**<br>Interaction: F=0.602, p=0.549
Plank duration (s)	F = 8.313, p =0.005**	F = 11.078, p =0.001***

$^{*}p < .05$, $^{**}p < .01$, $^{***}p < .001$,

TableS3. Impact of Enrollment Cohort on Motor Performance (COVID-19 Cohort Consistency Check).

**Measurement Items**	**Boys (*F, P*)**	**Girls*(F, P)***
25-meter dash (s)	$F = 0.841, p = 0.476$	$F = 0.425, p = 0.736$
Standing long jump (cm)	$F = 1.678, p = 0.180$	$F = 3.294, p = 0.025$*
Tennis ball throw	$F = 0.835, p = 0.479$	$F = 0.525, p = 0.666$
Double-leg continuous jump (s)	$F = 0.770, p = 0.515$	$F = 1.498, p = 0.222$
Plank duration (s)	$F = 0.574, p = 0.634$	$F = 0.952, p = 0.420$

*$p < .05$,

TableS4.Interaction Effects Between Gender and Early Exercise Experience on Motor Performance (Three-Way ANOVA).

**Measurement Items**	***F***	***P***
25-meter dash (s)	2.814	0.095
Standing long jump (cm)	2.133	0.146
Tennis ball throw	0.816	0.368
Double-leg continuous jump (s)	1.866	0.174
Plank duration (s)	2.607	0.108

*$p < .05$,
